# Importance of Revaccination

**Published:** 1871-05

**Authors:** 


Importance of Revaccination.—There is a case on record of a woman who was vaccinated, we believe by Jenner himself, who then resisted variolous inoculation, who some years after nursed her own children through the small-pox with impunity, and who, nevertheless, at a very advanced period of life, caught the disease from a grandchild, and died.—*Lancet.*

